# *SRSF2* Mutations in Uveal Melanoma: A Preference for In-Frame Deletions?

**DOI:** 10.3390/cancers11081200

**Published:** 2019-08-17

**Authors:** Natasha M. van Poppelen, Wojtek Drabarek, Kyra N. Smit, Jolanda Vaarwater, Tom Brands, Dion Paridaens, Emine Kiliç, Annelies de Klein

**Affiliations:** 1 Department of Ophthalmology, Erasmus University Medical Center, 3015 GD Rotterdam, The Netherlands; 2 Department of Clinical Genetics, Erasmus University Medical Center, 3015 GD Rotterdam, The Netherlands; 3 The Rotterdam Eye Hospital, 3011 BH Rotterdam, The Netherlands

**Keywords:** uveal melanoma, splicing, cancer, myelodysplastic syndrome

## Abstract

*Background:* Uveal melanoma (UM) is the most common primary ocular malignancy in adults in the Western world. UM with a mutation in *SF3B1*, a spliceosome gene, is characterized by three or more structural changes of chromosome 1, 6, 8, 9, or 11. Also UM without a mutation in *SF3B1* harbors similar chromosomal aberrations. Since, in addition to *SF3B1*, mutations in *U2AF1* and *SRSF2* have also been observed in hematological malignancies, UM without a *SF3B1* mutation—but with the characteristic chromosomal pattern—might harbor mutations in one of these genes. *Methods:* 42 UMs were selected based on their chromosomal profile and wildtype *SF3B1* status. Sanger sequencing covering the *U2AF1* (exon 2 and 7) hotspots and *SRSF2* (exon 1 and 2) was performed on DNA extracted from tumor tissue. Data of three UM with an *SRSF2* mutation was extracted from the The Cancer Genome Atlas (TCGA). *Results:* Heterozygous in-frame *SRSF2* deletions affecting amino acids 92–100 were detected in two UMs (5%) of 42 selected tumors and in three TGCA UM specimens. Both the UM with an *SRSF2* mutation from our cohort and the UM samples from the TCGA showed more than four structural chromosomal aberrations including (partial) gain of chromosome 6 and 8, although in two TCGA UMs monosomy 3 was observed. *Conclusions:* Whereas in myelodysplastic syndrome predominantly missense *SRSF2* mutations are described, the observed *SRSF2* mutations in UM are all in-frame deletions of 8–9 amino acids. This suggests that the R625 missense SF3B1 mutations and SRSF2 mutations in UM are different compared to the spliceosome gene mutations in hematological cancers, and probably target a different, as yet unknown, set of genes involved in uveal melanoma etiology.

## 1. Introduction

Uveal melanoma (UM) is a primary malignant ocular tumor arising from melanocytes in the uvea which consist of iris, ciliary body, and choroid. Symptoms are present in the majority of patients with the most common presenting symptom being change in vision. Other presenting symptoms include photopsia and floaters [1]. Metastatic disease with predominantly metastasis to the liver, develops in almost half of all UM patients causing a poor prognosis [1,2]. Several prognostic factors are described with mutations in *BAP1*, *SF3B1*, and *EIF1AX,* with or without loss of chromosome 3, as important predictors of survival [3,4]. Tumors of uveal melanoma (UM) patients with somatic *BAP1*, *SF3B1*, or *EIF1AX* mutations show a distinct chromosomal copy number variation (CNV) pattern. Whereas *EIF1AX*^mut^ tumors in general lack gross anomalies, *BAP1*^mut^ tumors display monosomy 3 and isochromosome formation. *SF3B1^mut^* tumors are characterized by three or more structural variants, usually of chromosomes 1, 6, 8, 9, and 11 [5]. However, not all UMs with a typical *SF3B1*^mut^ CNV harbor a mutation in the *SF3B1* component of the spliceosome complex. As in myelodysplastic syndrome (MDS) and MDS-related diseases (such as chronic myelomonocytic leukemia and acute myloid leukemia) in which mutations in other genes of the spliceosome complex such as *SRSF2* and *U2AF1* are described [6,7,8,9,10], mutations in *SRSF2* and other spliceosome factors are also observed in UM [11]. Typical MDS-related mutations in *SRSF2* involve codon 95 and are missense mutations resulting in an amino acid change (in 74% of patients with an *SRSF2* mutation) or in-frame deletions starting at this codon (26%) [8]. Missense mutations in *U2AF1* in MDS are almost exclusively described in codon 34 (p.Ser34Phe and p.Ser34Tyr), 156 (Arg156His), or 157 (p.Gln157Arg and p.Gln157Pro) [7,12]. Therefore, mutation analysis of *SRSF2* and *U2AF1* covering these hotspots was performed on UM tumors with no *SF3B1* mutation but with an *SF3B1*-like chromosomal CNV pattern.

## 2. Results

**Figure 1:**
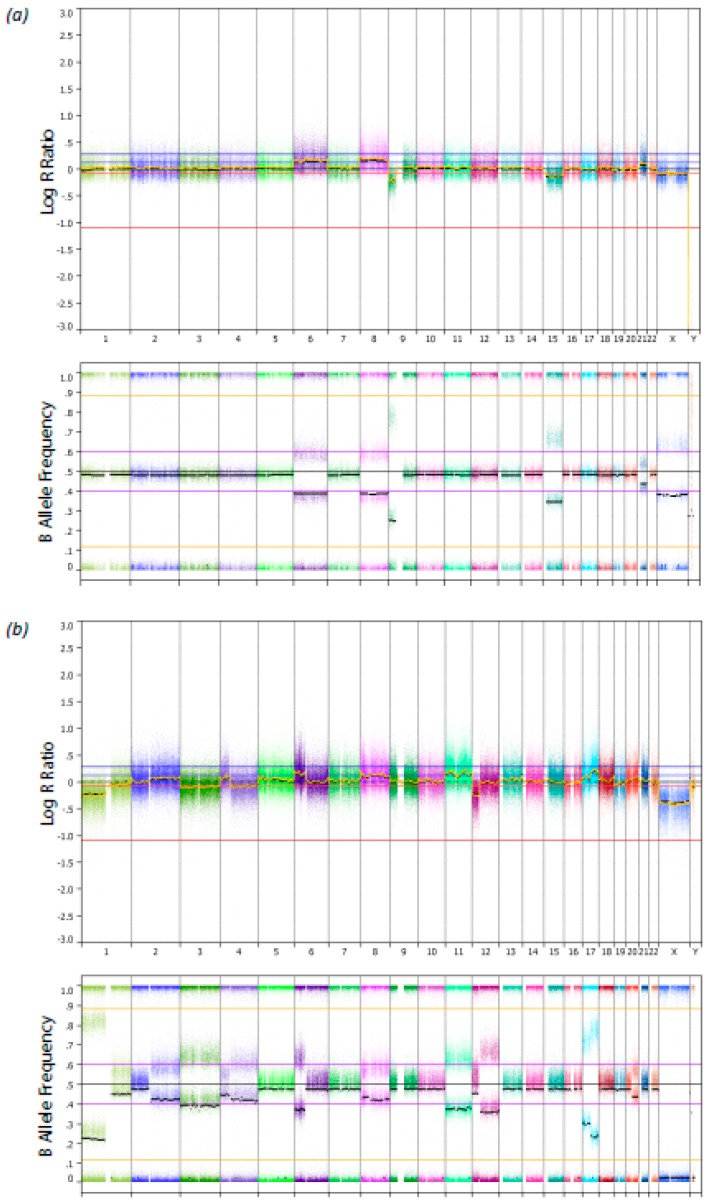
Single nucleotide polymorphism (SNP) array profile with the B-allele frequency from two uveal melanoma samples with an *SRSF2* mutation. On the x-axes the chromosomes are displayed. (**a**) Uveal melanoma 1 (UM1). (**b**) Uveal melanoma 2 (UM2).

Heterozygous in-frame deletions starting at codon 92 of *SRSF2* were identified in two of the selected 42 UM (p.(Tyr92_His99del); p.(Gly93_His100del)), (Figure 1). These mutations were mutually exclusive for *BAP1*, *SF3B1*, and *EIF1AX* but harbored a *GNAQ* p.(Gln209Leu) mutation (Table 1). 

UM1 originates from the ciliary body and consists of mixed cell type with the presence of closed vascular loops. Largest tumor diameter was 19 mm with a prominence of 8 mm. The other UM, UM2, arose from the choroid and consist of spindle cells. No closed vascular loops were present and there was no involvement of the ciliary body. The largest tumor diameter was 13 mm with a prominence of 5 mm with no extraocular extension.

**Figure 2:**
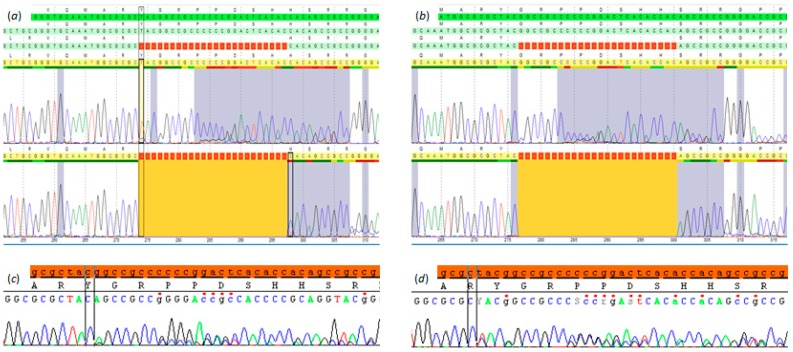
Mutations in the *SRSF2* gene from two out of the 42 analyzed uveal melanoma patients. (**a**) and (**c**) Uveal melanoma sample (UM1) with a p.Y92_H99del displayed in SeqPilot V4.3.0 (JSI medical systems, Ettenheim, Germany) (**a**) and in SeqScape V3.0 (Thermo Fisher Scientific, Waltham, MA, USA) (**c**). (**b**,**d**) Uveal melanoma (UM2) with a p.G93_H100del displayed in SeqPilot V4.3.0 (**b**) and in SeqScape V3.0 (**d**).

Both UMs showed more than four chromosomal aberrations including gain of chromosome 6 and 8. The single nucleotide polymorphism (SNP) array profiles of these tumors are shown in Figure 2. Both patients did not develop metastatic disease and have a disease-free survival of 76.8 and 128.8 months, respectively. In none of the 42 samples a mutation in *U2AF1* was detected. 

Three previously described *SRSF2* mutations were found in the data from the The Cancer Genome Atlas (TCGA) database [11]. CNV analysis showed loss of chromosome 3 in two UMs and gain of chromosome 8(q) in all three UMs. Gain of chromosome 1p was also present in two UMs and gain of chromosome 6 in one sample. Two UMs have a p.(Gln209Leu) mutation in *GNAQ* and one harbors a *GNA11* mutation (p.(Gln209Leu)), (Table 2). No mutations in *EIF1AX* were detected, but one UM has *BAP1* mutation (c.518A > G:p.(Tyr173Cys)). 

## 3. Discussion

In this study we identified deletions in *SRSF2* in two UM harboring an *SF3B1* specific SNP array pattern albeit with no mutations of the SF3B1 hotspot regions. Studies have shown that in myelodysplastic syndrome (MDS) *SRSF2* was mutated in 12–14% of the cases and mutations in *U2AF1* occur in 15% of the MDS cases [7,8]. This is a higher frequency compared to UM, in which *SRSF2* mutations are detected in less than 5% of the specimens and no *U2AF1* mutations have been identified [11]. Three *SRSF2* mutated UMs described in the literature are included in The Cancer Genome Atlas (TCGA, https://cancergenome.nih.gov/). Two out of these harbor similar deletions (Table 2) as we have identified in our cohort, and are mutually exclusive with *BAP1* and *EIF1AX*, similar to our own observations. The third *SRSF2* mutation from TCGA is a deletion of amino acid 174–179 and co-exists with a *BAP1* mutation. Surprisingly, this tumor showed a *BAP1* specific CNV profile, indicating that latter deletion of residues 174–179 has no or little pathogenic effect. However, other spliceosome gene mutations can underlie UM pathogenesis but might not display the same chromosomal anomalies as described in *SF3B1* [5]. Furthermore, the low incidence of *SRSF2* mutations in UM suggests that other genes of the splicing machinery, such as *U2AF35* or *ZRSR2,* might be mutated. Mutations in other splicing genes than *SF3B1* could be less frequently involved in the development of UM compared to MDS in which mutations in several splicing genes have been identified [6,9,10]. 

Since Sanger sequencing was used for mutation analysis, we have focused on the hotspot regions of *U2AF1* and *SRSF2* that are described in UM and other diseases. More extensive research about mutations in all coding regions of these genes could increase the incidence. 

Compared to *SRSF2* mutations in MDS in which the vast majority are missense mutations [8], we observed a preference for in-frame deletions in UM. Also, for SF3B1 in UM residue R625 is most commonly mutated residue, whereas in other tumors predominantly the K700 residue of SF3B1 is affected [6,11]. Thus, although the same gene is involved, mutations occur on different residues in distinct diseases. Furthermore, studying the RNA expression of *SRSF2* mutated UM from TGCA, we did not observe the same splicing effect as observed in *SF3B1* mutated UM. These findings suggest that *SF3B1* mutations compared to mutations in *SRSF2* have, despite a similar chromosomal pattern, a different effect on splicing.

Since we observed *SRSF2* mutations in only two patients the clinical impact of this mutation remains unclear. However, both patients with an *SRSF2* mutation in our cohort did not develop metastasis within 6 and 10 years, neither did the patients from TCGA. In chronic myelomonocytic leukemia no difference in overall survival was observed, and not in MDS when corrected for age [8,10]. Future studies are needed to evaluate the role of other splicing genes than *SF3B1* in UM.

## 4. Materials and Methods 

Patients with an *SF3B1*-like chromosomal pattern were selected from the Rotterdam Ocular Melanoma Study group (ROMS) database. These UM patients underwent enucleation or biopsy of the tumor in the Erasmus Medical Center (Rotterdam, The Netherlands) or The Rotterdam Eye Hospital (Rotterdam, the Netherlands) between 1993 and 2017. Informed consent from all patients was obtained before collecting the tumor material. This study was performed according to the tenets of the Declaration of Helsinki and approved by the local ethics committee (MEC-2009-375, 12th November 2009). 

DNA was isolated from fresh tumor tissue using the QIAamp DNA Mini Kit (Qiagen, Hilden, Germany) and concentrations measured using the Quant-iT PicoGreen dsDNA Assay Kit (Thermo Fisher Scientific, Waltham, MA, USA). Two hundred nanograms of DNA input was used for SNP-array analysis using an Illumina Human SNP array platform (Illumina, San Diego, CA, USA). Copy number analysis was performed using Nexus Copy Number 8.0 (BioDiscovery, El Segundo, CA, USA). Moreover, karyotyping was used for CNV analysis when available. Patients were selected from the cohort described previously [5].

In general, an *SF3B1*-like chromosomal pattern is defined as a combination of three structural variations in SNP array analysis of the tumor (usually this includes either partial gain of chromosome 8q or 9q or partial loss of chromosome 1p or 11q) [5]. In addition, UM with gain of chromosome 6p or loss of 6q in addition to one or two other anomalies were also included since these anomalies are also specific for *SF3B1* mutated tumors, whereas this is not seen in *EIF1AX* or *BAP1* mutated UM. Moreover, solely gain of chromosome 6p was only included when the tumor did not harbor an *EIF1AX* mutation, because gain of chromosome 6p is only representative for *SF3B1* and *EIF1AX* mutated UM. 

The two coding exons of the *SRSF2* gene were sequenced using Sanger sequencing with primers for these regions (pxlence, Dendermonde, Belgium). The mutation hotspots in *U2AF1* were sequenced with primers covering codon 34, 156, and 157. Sanger sequence results were visualized with SeqScapeSoftware V3.0 (Thermo Fisher Scientific, Waltham, MA, USA) and SeqPilot V4.3.0 (JSI medical systems GmbH, Ettenheim, Germany). 

Mutation analysis of *GNAQ*, *GNA11*, *SF3B1, BAP1*, and *EIF1AX* was performed previously using Sanger sequencing and next-generation sequencing using the ION Torrent platform (Life Technologies, Carlsbad, CA, USA) [5,13]. A *BAP1* mutation was defined as a mutation in the *BAP1* gene or lack of nuclear BAP1 expression (performed as described previously [14]). 

The UM cohort from the National Insitute of Health TCGA server (*n* = 80) was used for mutation analysis of *SRSF2* and *U2AF1* using Integrative Genomics Viewer (Version 2.3.68 (97) (Broad Institute, Cambridge, MA, USA). If a mutation in one of these genes was identified, copy number analysis was performed on the segmented SNP array data using Nexus Copy Number 8.0 (BioDiscovery, El Segundo, CA, USA). 

## 5. Conclusions

UMs harbor chromosomal aberrations correlated with their mutation status [5]. Mutations in SF3B1 and SRSF2, genes that are both involved in splicing, occur not only in UM but are described in MDS and MDS related diseases as well [8,9,10,11,15]. However, the mutation type in these genes are different in both diseases. In UM, SF3B1 is almost exclusively a missense mutation at residue 625 whereas in other diseases residue 700 is mutated [6,11]. In *SRSF2*, a different type of mutation is also observed in UM compared to MDS and MDS related diseases, but the same region is involved. We identified in-frame deletions of *SRSF2* in UM in the same genetic region, whereas most mutations in the same gene in MDS are missense mutations [8]. Therefore, we conclude that there might be a preference for in-frame deletions in *SRSF2* in UM when this gene is involved. We did not observe any mutation in *U2AF1* in our selected cohort, and the incidence of mutations of *SRSF2* is low. Although we have a selected cohort which might influence the incidence, this is in line with previous studies, in which no or few mutations in these genes are found in UM patients [11,16,17]. The clinical relevance of CNV pattern and the relation to spliceosome mutations remains unclear. More research is needed to evaluate the significance of these findings.

## Figures and Tables

**Table 1:** Overview of clinical characteristics, mutation status and copy number variation of uveal melanoma (UM) patients with an *SRSF2* mutation.

	UM 1	UM 2
Clinical characteristics		
Sex	Female	Male
Age at diagnosis (years)	63.0	57.3
Metastasis	No	No
Disease free survival (months)	76.8	128.8
Mutation status		
SRSF2	Chr17(GRCh37):g.74732946_74732969del c.274_297del:p.(Tyr92_His99del)	Chr17(GRCh37):g.74732943_74732966del c.277_300del:p.(Gly93_His100del)
U2AF1	Wildtype	Wildtype
GNAQ	Chr9(GRCh37):g.80409488T > A c.626A > T:p.(Gln209Leu)	Chr9(GRCh37):g.80409488T > A c.626A > T:p.(Gln209Leu)
GNA11	Wildtype	Wildtype
SF3B1	Wildtype	Wildtype
BAP1	Wildtype	Wildtype
EIF1AX	Wildtype	Wildtype
Copy number variation		
(Partial) gain of chromosome	6, 8, 21	2q, 6p, 8, 11, 17, 20q
(Partial) loss of chromosome	9p, 15	1p, 3, 4q, 12p

**Table 2:** Overview of mutations in uveal melanoma samples with an *SRSF2* or *U2AF1* mutation. ROMS = Rotterdam Ocular Melanoma Studygroup; TCGA = The Cancer Genome Atlas.

	*SRSF2*	*U2AF1*	*GNAQ*	*GNA11*	*BAP1*	*EIF1AX*
**1. ROMS**	c.274_297del:p.(Tyr92_His99del)	Wildtype	c.626A > T:p.(Gln209Leu)	Wildtype	Wildtype	Wildtype
**2. ROMS**	c.277_300del:p.(Gly93_His100del)	Wildtype	c.626A > T:p.(Gln209Leu)	Wildtype	Wildtype	Wildtype
**3. TCGA**	c.274_297del:p.(Tyr92_His99del)	Wildtype	c.626A > T:p.(Gln209Leu)	Wildtype	Wildtype	Wildtype
**4. TCGA**	c.274_300del:p.(Tyr92_His100del)	Wildtype	Wildtype	c.626A > T:p.(Gln209Leu)	Wildtype	Wildtype
**5. TCGA**	c.519_536del:p.(Ser174_Ser179del)	Wildtype	Wildtype	c.626A > T:p.(Gln209Leu)	c.518A > G:p.(Tyr173Cys)	Wildtype
